# Application of a Simplex–Centroid Mixture Design to Evaluate the Phenolic Compound Content and Antioxidant Potential of Plants Grown in Mexico

**DOI:** 10.3390/foods12183479

**Published:** 2023-09-19

**Authors:** Ricardo Omar Navarro-Cortez, Yair Olovaldo Santiago-Saenz, César Uriel López-Palestina, Jorge Gutiérrez-Tlahque, Javier Piloni-Martini

**Affiliations:** 1 Department of Agroindustrial Engineering and Food Engineering, School of Forestry and Environmental Studies, Universidad Autonoma del Estado de Hidalgo, Rancho Universitario Av. Universidad km 1, Ex-Hacienda de Aquetzalpa, Tulancingo 43600, Hidalgo, Mexico; ricardo_navarro@uaeh.edu.mx (R.O.N.-C.); javier_piloni7632@uaeh.edu.mx (J.P.-M.); 2 Department of Nutrition, School of Medical Sciences, Universidad Autonoma del Estado de Hidalgo, Circuito Ex Hacienda, La Concepción, Carretera Pachuca Actopan, San Agustin Tlaxiaca 42160, Hidalgo, Mexico; 3 Department of Food Engineering, Zitacuaro Institute of Technology, Av. Tecnológico Manzanillos, No. 186, Zitacuaro 61534, Michoacan, Mexico; jorge.gt@zitacuaro.tecnm.mx

**Keywords:** mixtures, endemic foods, sustainability, nutritional value

## Abstract

Nowadays, the food and health industries are generating new products with antioxidant potential; among them are those rich in phenolic compounds that have a beneficial impact on human health. Therefore, the aim of this research was to obtain different types of mixtures from *Portulaca oleraceae* (P), *Chenopodium album* (C), *Opuntia oligacantha* Förster var. Ulapa (O), and *Amaranthus tricolor* (A) and evaluate the content of total phenols, total flavonoids, and antioxidant potential in order to select the mixture with the highest content of phenolic compounds. An experimental simplex–centroid mixture design with 15 experimental treatments was used; the data were analyzed and adjusted to a quadratic model that allowed for the prediction of the content of phenols, flavonoids, and antioxidant activity using 2,2-diphenyl-1-picrylhydrazyl (DPPH) and 2,2′-Azino-bis (3-ethylbenz-thiazoline-6-sulfonic acid) (ABTS) of different experimental mixtures. The results show that the individual components of C and P had the highest content of phenols and antioxidant potential. It was observed that the binary mixtures P-C and P-A presented values of total phenols greater than 11 mg of gallic acid equivalents g^−1^ DW and values of flavonoids greater than 13 mg of quercetin equivalents g^−1^ DW. These values were higher than those found in the individual components. The P-C mixture with an antioxidant potential of 66.0 ± 0.07 Trolox equivalents g^−1^ DW could be used as an additive in food or to obtain a functional food that improves the intake of antioxidant compounds in the population.

## 1. Introduction

The phytochemicals found in foods of plant origin (fruits and vegetables) have been widely studied due to their ability to neutralize free radicals [1,2]. These compounds have the ability to donate hydrogen atoms or electrons to neutralize reactive oxygen species (ROS) or nitrogen (RNS), which are oxidizing species of cells and other biological components [3]. Therefore, these compounds are of importance for the survival of plants and animals. Plants synthesize phenolic compounds (PCs) to function as a chemical defense against predators, as protection against ultraviolet light, as pollinator attractants, and as participants in reproduction as well as in plant–plant interference [4,5]. On the other hand, in humans, they continue to be useful in the fields of health and nutrition due to the health benefits of their dietary intake, mainly protection against chronic diseases, inflammatory states, and dysfunctional mechanisms associated with oxidative stress [1,4,6,7,8,9]. As an example, a lower risk of suffering a myocardial infarction, diabetes, and a cerebral vascular accident, as well as improvements in blood flow and blood pressure, among others, have been associated with the consumption of the flavan-3-ols found in cocoa, while resveratrol and quercetin improve cardiometabolic health. Some studies mention that phenolic compounds are metabolized in the body and by the intestinal microbiota, generating structural modifications in the compounds or conjugations of compounds and generating new molecules that are beneficial for human health [10].

However, not everyone can have access to the quality, quantity, and bioavailability of phenolic compounds due to various factors, including the high consumption of fast food that is characterized by low nutritional value and high caloric intake, which is popular among people with a fast-paced lifestyle. Food preferences or unhealthy eating habits can lead to an increase in non-communicable diseases. On the other hand, in various regions, vegetables and fruits are consumed after having undergone some type of heat treatment, which affects the quality of bioactive compounds [11].

Based on this evidence, some researchers have worked to promote the consumption of diets rich in these compounds [4,9]. Others have highlighted the importance of generating clear and regulated alternatives in the food industry through their incorporation into foodstuffs, nutraceutical formulations, and the production of dietary supplements. These can be produced in different forms, such as capsules, powders, or mixtures of natural origin, that allow an increase in the content of these phytochemicals [1,7]. These supplements have been used for various pathological or physiological conditions, serving as a support for the diet that lacks these compounds (the supplement does not substitute a mealtime), and good results have been obtained in various studies with animals and humans. For example, in an intervention using grape seed supplements rich in phenolic compounds, researchers observed a significantly reduced (33%) urine redox potential, reflecting an overall increase in the antioxidant status of the volunteers [12]. On the other hand, the literature has reported an increase in urinary arsenic [13] and fluorine [14] excretion using supplements of green leafy vegetables rich in phenolic compounds. In another study, the power of antioxidants and their benefits for athletes was described, taking into account the optimal dosing methods. According to this research, a suggested dosage of not greater than 1 g/day and other considerations established by evidence should be respected [15]. Despite being a debatable topic, the research shows that the use of antioxidant-polyphenol-rich dietary supplements or other kinds of presentations can upregulate the endogenous antioxidant defense system, preventing excessive oxidative damage [15].

A viable, economical, and PC-rich alternative source is Mexican plants. In Mexico, a number of leafy vegetables are consumed, as well as some fruits, that are a source of vitamins, phenolic compounds, and minerals [6,8,16]. Among the leafy vegetables consumed mainly in central Mexico is the species *Portulaca oleracea*, known as purslane, which is rich in flavonoids (quercetin, kaempferol, myricetin) [17,18]; the genera *Chenopodium* spp., among which we identify the ashen quelite, quinhuilla, white quelite, donkey quelite, huazontle, and epazote and the genera *Amaranthus* spp., including the white, red, or tricolor quintonil, which are all rich in caffeic acid, gallic, vanillic, syringic, *p*-hydroxybenzoic, and ferulic [18]; and finally, some fruits of the genera *Opuntia*, such as *Opuntia oligacantha* var. Ulapa (xoconostle), is described as a rich source of PCs such as gallic, syringic, and ferulic acids, among others [16,19,20,21]. As mentioned above and according to references, these plant species are rich sources of phenolic compounds and can serve as cheap raw materials to obtain food supplements rich in these phytochemicals, providing an alternative for use in the food industry and a complementary alternative within the diet. Therefore, the aim of this research was to evaluate the content of phenols, flavonoids, and antioxidant activity by DPPH and ABTS of mixtures obtained from three leafy vegetables (*Portulaca oleracea*, *Chenopodium album*, and *Amaranthus tricolor*) and a fruit (xoconostle) native to the state of Hidalgo, Mexico.

## 2. Materials and Methods

### 2.1. Reagents and Chemicals

2,2′-Diphenyl-1-picrylhydrazyl (DPPH), 2,2′-azino-bis(3-ethylbenzothiazoline-6-sulfonic acid) (ABTS), Trolox (6-hydroxy-2,5,8-tetramethylchroman-2-carboxylic acid), and Folin–Ciocalteau reagent were purchased from Sigma-Aldrich (St. Louis, MO, USA). Sodium hydroxide, sodium carbonate, aluminum chloride, and potassium sulfate were purchased from J.T. Baker (Bridgewater, NJ, USA). Gallic acid, quercetin, methanol, and ethanol were purchased from Meyer Chemical (Tlahuac, Mexico); additionally, potassium persulfate was acquired from Reasol laboratories (Mexico City, Mexico). Sodium nitrite was purchased from Monterrey Chemical Products (Monterrey, Mexico). Distilled water was used in the performance of all the experiments, and all the reagents used were of analytical grade.

### 2.2. Plant Material

Samples of *Portulaca oleracea* (wild purslane or quelite de Monte), *Amaranthus tricolor* (wild quintonil tricolor), and *Chenopodium album* (wild quinhuilla) were collected in the municipality of Santiago de Tulantepec, Hidalgo, Mexico (latitude: 19°59′ and 20°05′ N, longitude: 98°20′ and 98°29′ W). The samples were collected in April 2023. The fruits of *Opuntia oligacantha* (Förster) variety Ulapa were obtained from the region of Ulapa de Melchor Ocampo, Hidalgo, Mexico (latitude: 19°53′00″ N, longitude: 98°49′00″ W). The roots were removed from the plants, leaving only the aerial parts (leaves). Regarding the fruit of *Opuntia oligacantha* (Förster) var. Ulapa, the epicarp and mesocarp were used. The plants and the fruit were identified at the herbarium of the Universidad Autonoma del Estado de Hidalgo (collector: Santiago-Saenz, Y.O; identification numbers of P-W1, C-W2, O-W3, A-W4).

### 2.3. Selection and Preparation of Plant Material

The first step was obtaining disease-free plant material (leaves). Then, the leaves were washed, rinsed with distilled water, and dried for 1 h at room temperature (25 °C). The leaves of the plant materials were placed in hermetically sealed polyethylene bags and stored at −76 °C in an ultra-low temperature freezer (Thermo-Scientific, 703, Outside, Waltham, MA, USA) for one week to facilitate the subsequent freeze-drying process. The xoconostle fruits were washed and rinsed with distilled water, and then the endocarp was separated from the fruit, as this part was not used in this research. The epicarp and mesocarp of the fruit were analyzed together because they are the parts that are most consumed. They were placed in hermetically sealed polyethylene bags and stored at −76 °C in an ultra-low-temperature freezer (Thermo-Scientific, 703, Outside, Waltham, MA, USA) for one week. Subsequently, the samples were dried in a Labconco freeze dryer (Labconco, Model 7948000, St. Louis, MO, USA) at 133 × 10^−3^ mBar and −40 °C; once dry, they were ground in a Grindomix mill (Retsch, GM 200, Newton, PA, USA) at 9000 rpm for 1 min. The particle size of the obtained powder was 150 μm, according to equipment specifications. The powders obtained from the lyophilized plant materials were mixed according to the experimental design.

### 2.4. Experimental Design

A simplex–centroid mixture design of experiments [22] was used. The independent variables were proportions of powders from *Portulaca oleracea*, *Chenopodium album*, *Opuntia oligacantha* Förster var. Ulapa, and *Amaranthus tricolor.* The coded values of independent variables are shown in Table 1. The experimental design resulted in 15 experimental mixtures. The coded value 1 indicates the presence of a single plant material (individual component); the value 0.5 indicates a mixture with two plant materials in equal proportion (binary mixture); the value 0.3 indicates a mixture with three plant materials in equal proportion (ternary mixture); and the value 0.25 indicates a mixture with four plant materials in equal proportion (quaternary mixture). The dependent variables evaluated for each mixture were the content of phenols and flavonoids, and the antioxidant activity determined by DPPH and ABTS. The experimental data were adjusted to a second-order model (Equation (1)) using the statistical package Design Expert version 7.1.6 (Statease, Minneapolis, MN, USA). The fit of the model to the data was evaluated by ANOVA. The general expression of the model was the following:

y = *β*_1_*X*_1_ + *β*_2_*X*_2_ + *β*_3_*X*_3_ + *β*_4_*X*_4_ + *β*_1,2_*X*_1_*X*_2_ + *β*_1,3_*X*_1_*X*_3_ + *β*_1,4_*X*_1_*X*_4_ + *β*_2,3_*X*_2_*X*_3_ + *β*_2,4_*X*_2_*X*_4_ + *β*_3,4_*X*_3_*X*_4_ (1)

where:

y: response
*β*_i_: regression coefficients of component *X*_i_ (the coefficient has no units)
*X*_1_: *Portulaca oleracea*
*X*_2_: *Chenopodium album*
*X*_3_: *Opuntia oligacantha* Förster var. Ulapa
*X*_4_: *Amaranthus tricolor*

**Table 1:** Simplex-centroid experimental design with four components: *X*_1_ (*Portulaca Oleracea*), *X*_2_ (*Chenopodium album*), *X*_3_ (*Opuntia oligacantha* Förster var. Ulapa), and *X*_4_ (*Amaranthus tricolor*).

	Mixture	*X*_1_	*X*_2_	*X*_3_	*X*_4_
Individual components	1	1.0	0.0	0.0	0.0
	2	0.0	1.0	0.0	0.0
	3	0.0	0.0	1.0	0.0
	4	0.0	0.0	0.0	1.0
Binary mixture	5	0.5	0.5	0.0	0.0
	6	0.5	0.0	0.5	0.0
	7	0.5	0.0	0.0	0.5
	8	0.0	0.5	0.5	0.0
	9	0.0	0.5	0.0	0.5
	10	0.0	0.0	0.5	0.5
Ternary mixture	11	0.33	0.33	0.33	0.0
	12	0.33	0.33	0.0	0.33
	13	0.33	0.0	0.33	0.33
	14	0.0	0.33	0.33	0.33
Quaternary mixture	15	0.25	0.25	0.25	0.25

### 2.5. Quantification of Total Phenol and Flavonoid Content

From the mixtures obtained, 0.5 g samples were taken and homogenized with 10 mL of an ethanol–water solution (80:20 *v*/*v*), after which they were placed in an ultrasonic bath (Mod. 32V118A, Freeport, IL, USA) for 15 min at a frequency of 40 kHz at room temperature, and finally, they were centrifuged (Thermo-Scientific, ST 16R, Waltham, MA, USA) at 16,500× *g* for 10 min, and the supernatant was collected for further analysis.

Total phenols were determined spectrophotometrically according to the method of Rosales et al. [23]. An amount of 0.5 mL of the supernatant was taken and mixed with the Folin–Ciocalteu reagent (50% *v*/*v* in distilled water), 2% (*w*/*v*) sodium carbonate solution, and distilled water. The mixture was left to stand in the dark for 60 min before the absorbance at 725 nm was determined on a spectrophotometer (model 6715 UV/Vis, Jenway, Techne Inc., Stone, Staffordshire, UK). The total phenol content was reported as mg gallic acid equivalents (mg GAE) per g of the dry weight of raw material (DW).

The flavonoid content was determined according to Rosales et al. [23]. A total of 0.5 mL of the supernatant was mixed with distilled water and NaNO_2_ (5% *w*/*v*), and the mixture was left to rest for 5 min. Then, AlCl_3_ and the 1 M NaOH solution were added, and the absorbance was measured at 415 nm. The total flavonoid content was reported as mg quercetin equivalents (mg QE) per g of the dry weight of raw material (DW).

### 2.6. Antioxidant Potential

#### 2.6.1. Determination of Antioxidant Potential by DPPH

The determination was made according to the method described by Brand-Williams et al. [24]. An amount of 0.5 mL of the supernatant was reacted with the DPPH^•+^ radical with a concentration of 6 × 10^−5^ mM. The mixture was left to stand in the dark for 60 min at 4 ± 1 °C, and, subsequently, the absorbance at 515 nm was determined. Results were expressed as µM Trolox equivalents (µM TE) per g of the dry weight of raw material (DW).

#### 2.6.2. Determination of Antioxidant Potential by ABTS

The ABTS^•+^ radical was obtained by reacting the ABTS reagent at a concentration of 7.0 mM with potassium persulfate at a concentration of 2.45 mM at room temperature (±25 °C) for 16 h [25]. Once the ABTS^•+^ radical was formed, it was mixed with 0.5 mL of the supernatant. The mixture was left to rest for 6 min in the dark, and the absorbance was measured at 734 nm. Results were expressed as µM Trolox equivalents (µM TE) per g of the dry weight of raw material (DW).

### 2.7. Analysis of the Results

Experimental data are presented as means ± standard deviation (experiments in triplicate). A one-way ANOVA was performed to find statistically significant differences between the individual components and the mixtures using Tukey’s mean comparison test (*p* ≤ 0.05). A Pearson correlation was performed to find the degree of linear association between the dependent variables using the Statistica 10.0 statistical package (StatSoft Inc., Tulsa, OK, USA).

## 3. Results and Discussion

### 3.1. Regression Coefficients of the Fitted Model for the Evaluated Responses

Table 2 shows the multiple linear regression coefficients of the adjusted quadratic model for total phenols, total flavonoids, and antioxidant activity by DPPH and ABTS. Regression coefficients in bold indicate a significant statistical effect (*p* ≤ 0.05) on the evaluated variables. A factor was considered to have a significant effect on the response if the value was found to be less than 0.05 (95% confidence level) [26]. Coefficients with a positive sign indicate an increase in the evaluated variable, while coefficients with a negative sign indicate a decrease in the evaluated variable. The adjusted R^2^ for the different responses was between 0.93 and 0.98; therefore, the quadratic model was valid in explaining the experimental data of the evaluated variables [27]. The individual components presented a significant positive statistical effect on all the variables evaluated. The interaction coefficients indicate a synergistic or antagonistic effect on the response variables when there is more than one component in the mixture [28]. The interaction coefficients indicate that the mixture of *Portulaca oleracea* with *Chenopodium album* (*β*_1_*β*_2_) presented a synergistic effect for DPPH and an antagonistic effect for ABTS. On the other hand, it was found that the mixture of *Portulaca oleracea* with *Opuntia oligacantha* Förster var. Ulapa (*β*_1_*β*_3_) presented a synergistic effect for the content of total phenols, total flavonoids, and antioxidant activity for ABTS. Meanwhile, the mixture of *Portulaca oleracea* with *Opuntia oligacantha* Förster var. Ulapa (*β*_1_*β*_4_) presented a significant synergistic effect for the responses of total phenols, total flavonoids, and antioxidant activity by DPPH. Regarding the mixture of *Chenopodium album* with *Opuntia oligacantha* Förster var. Ulapa (*β*_2_*β*_3_), a significant antagonistic effect on the responses of total phenols, total flavonoids, and antioxidant activity by ABTS was observed. Concerning the mixture of *Chenopodium album* with *Amaranthus tricolor* (*β*_2_*β*_4_), a significant antagonistic effect for total phenols, total flavonoids, and antioxidant activity by ABTS was found, while a synergistic effect was presented for antioxidant activity by DPPH. Finally, the mixture of *Opuntia oligacantha* Förster var. Ulapa with *Amaranthus tricolor* (*β*_3_*β*_4_) showed a significant antagonistic statistical effect for total flavonoids and antioxidant activity by ABTS but did not present a significant statistical effect for the total phenol responses or DPPH. The impact of different mixtures on DPPH and ABTS may be because the DPPH radical does not react so easily with flavonoids, given that they are molecules with a higher molecular weight, which makes access to the DPPH radical difficult for its subsequent reduction. Additionally, the reduction potential of the DPPH radical is −1.2 V, whereas for the ABTS radical it is −0.67 V; therefore, ABTS has the advantage of being more reactive and thus likely has the ability to react with a broader range of antioxidants present in plant-based mixtures. In addition, steric accessibility also plays an important role in reactivity; molecules without large substituents have better access to the radical, which is important in relation to DPPH [29,30,31].

### 3.2. Total Phenol Content

Table 3 shows the total phenol content of the individual components and the binary, ternary, and quaternary mixtures. The content of total phenols was in the range of 6.6 ± 0.05 to 11.3 ± 0.02 mg equivalents of gallic acid (GAE) per g of dry weight (DW). Of the individual components, *Portulaca oleracea* (P) and *Chenopodium album* (C) had the highest content of total phenols, with concentrations greater than 10 mg GAE g^−1^ DW, while *Opuntia oligacantha* Förster var. Ulapa (O) and *Amaranthus tricolor* (A) presented the lowest content of total phenols (Table 3). *Portulaca oleracea–Chenopodium album* (P-C), *Portulaca oleracea–Amarantus tricolor* (P-A), and *Portulaca oleracea–Opuntia oligacantha* Förster var. Ulapa (P-O), as well as the ternary mixture *Portulaca oleracea–Opuntia oligacantha* Förster var. Ulapa–*Amaranthus tricolor* (P-O-A) presented values for total phenols above the individual components, while the ternary mixture *Portulaca oleracea–Chenopodium album–Opuntia oligacantha* Förster var. Ulapa (P-C-O) and the quaternary mixture (P-C-O-A) did not present significant statistical differences when compared with the individual components of *Portulaca oleracea* and *Chenopodium album.*

The results found in the different mixtures show that the content of total phenols can be increased by mixing vegetables rich in bioactive compounds in different proportions. In this regard, Santiago-Saenz et al. [32] reported values of total phenols similar to this study in binary mixtures of Mexican quelites of other species, such as *A. hybridus* and *C. berlandieri.* On the other hand, the concentrations of the individual components were very similar to those reported by other authors. Uddin et al. [33] reported values of 2.76 mg GAE g^−1^ for total phenols in *Portulaca oleracea* cultivated in Malaysia, obtaining the highest content of phenolic compounds in mature plants; Arias-Rico et al. [34] reported higher values than those obtained in this work for total phenols in *Portulaca oleracea* (14.77 ± 0.883 mg GAE g^−1^). On the other hand, Siriamornpun and Suttajit [35] reported that the highest content of total phenols in *Portulaca oleracea* was found in the leaves, which is consistent with this study when using this portion of the samples.

Regarding the fruit, Hernández-Fuentes et al. [20] reported lower values for total phenols in *Opuntia oligacantha* pulp (2.78 ± 0.02 mg GAE g^−1^), while Gómez-Covarrubias et al. [36] reported higher values for total phenols (9.2 ± 0.81 mg GAE/mL) in xoconostle juice of the joconostle variety. It is worth noting that the fruit in our research presented a considerable variety of phenolic compounds, which were comparable to those that have been reported in the literature for other varieties (Table S1). The phenolic content can also vary between plants and fruits. This is mainly due to differences in climatic conditions, soil type, water supply, and stage development of the plant [19], as well as the methods used for its extraction [37]. However, it should be noted that both the weed species and the wild fruit analyzed presented promising contents of phenols and flavonoids, which can be increased and presented with greater variability when making specific ternary mixtures.

Additionally, according to the results of this investigation, the phenol content was greater in the mixtures where *Portulaca oleracea* was present. This is due to the phenolic profile that precedes this plant [18,35]. Some studies have shown the antioxidant and anti-inflammatory capacity of this genera, reporting its great ability to counteract the effects of chronic diseases caused by oxidative stress due to its antioxidant effect, which is attributed to the high content of phenolic acids and flavonoids found in the stems and leaves of its respective species [8,35,38,39]. In addition, other studies have reported that by incorporating purslane into the diet of egg-laying hens, egg production and weight can be increased without changes in the cholesterol concentration of the product, which is why it is proposed as a vegetable alternative for the enrichment of livestock products [40]. This plant is considered a promising natural product and has been promoted as a complementary addition to human nutrition [35]. Purslane, being an easy-to-find and readily available resistant plant with high nutritional value, has been proposed for the elaboration of functional foods and nutraceutical applications [33,41]. The evidence has shown that when purslane and other quelites are incorporated with other raw materials, such as wheat, or added to different products, such as juices, tomato sauces, or yogurt, they can improve the sensory and technological characteristics and the antioxidant content [42].

Moreover, according to Román-Cortés et al. [43], the genera *Chenopodium* spp., which was also included in the mixtures, tends to show high phenol and flavonoid contents compared to other species of Mexican quelites. *Chenopodium* has been recognized for its high nutritional value and phenolic components that are associated with its beneficial effects on health, such as its antioxidant and anti-inflammatory properties, proposing itself as a potential source for new foods that can be used by the food industry [44,45]. Based on the evidence, the combination of these genera of plants with legumes or as an ingredient (dried leaves or powders) with other raw materials for the production of snacks, bread, noodles, and fermented products can improve protein profiles and the content of phytochemicals (PCs) with high antioxidant activity more than using other common vegetables such as cabbage and contribute to better immune function, among other biological effects [44,45]. On the other hand, the genera *Amaranthus* spp., incorporated in mixture 7, have also shown considerable phenolic profiles and antioxidant properties, which is why they are considered foods rich in phenolic compounds, displaying great potential for their incorporation into functional foods and nutraceuticals [43,46,47]. Likewise, the aerial parts of these plants have been proposed for the elaboration of food products, together with other raw materials, for the creation of snacks or drinks due to their phytochemical potential, and as an adjuvant for the prevention and control of chronic diseases [42,46]. In general, plant mixtures have been previously suggested by the literature to increase the content of proteins and phenolic compounds to contribute to the mechanisms that counteract oxidative stress or improve the health status of the patient [42,45,48].

### 3.3. Flavonoids Content

In Table 3, it can be seen that the highest content of flavonoids in the individual components was found in *Chenopodium album*, followed by *Amaranthus tricolor* and *Portulaca oleracea*, while the lowest flavonoid content was presented by *Opuntia* oligacantha Förster var. Ulapa. Regarding the binary mixtures, the highest content was presented by the P-C mixture, followed by P-A and C-A, where it was observed that *Chenopodium album* and *Amaranthus tricolor* were the main raw materials that increased the flavonoids in the mixtures obtained. This was confirmed in the P-C-A ternary mixture, which presented the highest total flavonoid content with values higher than 13 mg QE g^−1^ DW; hence, the results were consistent with what was observed by Román-Cortés et al. [43] in plants of the same genera but with different species. However, both plants are considered potential raw materials. It is important to highlight that the P-C mixture occupied the third position, with a considerable value of flavonoids, as *Portulaca oleracea* was included in the combination, which has been described as an excellent vegetable source rich in these phytochemicals [42]. On the other hand, when the leaves of the vegetables and the fruit of xoconostle were mixed in equal proportions, the quaternary mixture presented a lower flavonoid content. Santiago-Saenz et al. [18] report similar values for total flavonoids in *Portulaca oleracea*, *Chenopodium berlandieri*, and *Amaranthus hybridus* (8–15 mg QE g^−1^ DW). They also identified that these values are usually high in these plants due to the variety of flavonoids found, such as myricetin and apigenin (*Portulaca oleracea*), rutin, phloridzin, myricetin, quercetin, phloretin (*Chenopodium berlandieri*), and rutin and phloridzin (*Amaranthus hybridus*). Regarding the fruit, Gómez-Covarrubias et al. [36] reported lower values for total flavonoids in xoconostle var. joconostle, while López-Palestina et al. [16] reported slightly higher values (4.6 mg QE g^−1^ DW) in samples of the same variety of xoconostle. This difference may be due to the harvest seasons, which may influence the variability of the results [19]. Therefore, when obtaining mixtures rich in phenolic compounds with a certain content, it is necessary to take into account the harvest season of the raw materials. In addition, the values obtained for flavonoids were higher in some cases than phenols, both for the individual components and for some mixtures (Table 3), which is possibly due to the presence of other antioxidant compounds, such as proanthocyanidins, as has been observed in some quelites [43].

Additionally, some studies have mentioned the importance of foods of plant origin due to their contribution of polyphenols and flavonoids; however, due to the perishable nature of fruits and vegetables, their conversion into powders helps to preserve the phytochemical and nutritional components and gives them a relevant role in determining the texture, color, and sensory properties when formulating a food product, including a variety of foods such as soups and drinks, among others [49]. Ying et al. [49] evaluated two vegetables characterized by a rich content of phytochemicals (broccoli and carrot) in their powder presentation and obtained good results for the flavonoid content. Compared to our study, it was observed that the P-C mixture contained two-fold as many flavonoids. For this reason, the incorporation of this powdered mixture into the production of food is viable, on the one hand, to improve the variety of phenols and flavonoids (P-C) and, on the other hand, to promote the stability of these antioxidant compounds in the products.

### 3.4. Antioxidant Potential by DPPH and ABTS

Table 3 shows the results for antioxidant potential by DPPH and ABTS of the different mixtures obtained. The antioxidant activity for DPPH was in the range of 21.4 to 27.7 µmol TE g^−1^ DW; *Chenopodium album* presented the lowest antioxidant activity among the individual components, while *Opuntia oligacantha* Förster var. Ulapa presented the highest antioxidant activity. Among the binary mixtures, P-O presented the highest value for antioxidant activity, followed by O-A. In the ternary mixtures, P-C-O and P-O-A presented the highest antioxidant activity. For the quaternary mixture, in which there were 0.25 parts of each component, the antioxidant activity was superior at 25 µmol TE g^−1^ of sample DW. Similar values regarding the antioxidant activity of DPPH were reported for the type *Chenopodium berlandieri* [18] and the genera *Opuntia* [50]. On the other hand, the antioxidant activity measured by the ABTS assay was in the range of 43.4 ± 0.39 to 74.6 ± 0.25 µmol TE g^−1^ DW for individual components, from 38.7 ± 0.28 to 68.9 ± 0.07 µmol TE g^−1^ DW for binary mixtures, from 34.4 ± 0.26 to 57.3 ± 0.14 µmol TE g^−1^ DW for ternary mixtures, and the quaternary mixture presented a value of 51.3 ± 0.12 µmol TE g^−1^ DW. The individual components *Portulaca oleracea* and *Chenopodium album* presented the highest values without presenting a statistical difference between them, which explains the high antioxidant potential in the binary mixtures P-O, P-C, and P-A. According to Santiago-Saenz et al. [18], similar behaviors could be observed in the same genera of plants but with different species. Regarding antioxidant potential values in xoconostle, the literature reports similar anti-radical activities for ABTS (15–67 µM TE g^−1^ DW) in *Opuntia* spp. (*O. ficus-indica*, *O. stricta*, *O. undulata*) [50]. In the literature, antioxidant potential values have been reported in three varieties of xoconostles that are lower than those reported in this study (11–12 µM TE g^−1^ DW for ABTS) [20], indicating that the Ulapa variety has potential as a raw material to increase the antioxidant potential in food. In this sense, the individual component of xoconostle and the mixtures that incorporate this fruit (P-O) displayed better antioxidant activity by DPPH. This is mainly due to the various phenolic compounds with antioxidant capacity (Table S1) with which *Opuntia* has been related; therefore, although the phenol and flavonoid content did not show the highest values, the fruit may present other compounds with antioxidant activity such as betalains (nitrogenous anthocyanins), which are typical of this fruit [16,51] and may increase antiradical activity when combined with other plants rich in antioxidant compounds, such as *Portulaca*.

Regarding the antioxidant activity determined by ABTS, the values obtained increased to almost double those quantified by DPPH. This is mainly because the DPPH radical does not react with flavonoids due to the larger size of these molecules or with aromatic acids that have a single OH group; meanwhile, the ABTS assay reacts with both lipophilic and hydrophilic antioxidant compounds [52]. This could also explain the high values of the antioxidant potential in the cases of *Portulaca* by ABTS assay in the individual component and in the mixture, since the ABTS radical could quantify the antioxidant potential of polyunsaturated fatty acids and lipophilic antioxidants identified in its structure, such as tocopherols, in addition to the phenolic compounds evaluated in this study [42]. On the other hand, the potential of *Chenopodium* as an antioxidant is particularly due to the presence of polyphenols, mainly those derived from flavonoids (Table 3) that behave as reducing agents, mostly in the form of hydrogen donors and singlet oxygen quenchers [43,45]. Therefore, the evidence supports the use of these species to fortify the antioxidant potential in various foods or as supplements rich in natural antioxidants to prevent oxidative stress, a huge problem that has become a major contributor to an unhealthy lifestyle, which has been linked to a number of diseases. In this sense, supplementation with species rich in antioxidant compounds becomes another alternative for the formulation of mixtures in the preparation of nutraceuticals and supplements. Supplementation studies have shown that the increase in antioxidant levels through the use of powders, mixtures, or formulations can be positive, improving or delaying the worsening of the health status of patients with diseases related to oxidative stress [53]. Some investigations have studied foods rich in antioxidant compounds, such as strawberries [54], mixed fruit, vegetable juice concentrates [55], and prickly pears [56], reporting a significant increase in the total antioxidant capacity of plasma due to the levels of important antioxidants that positively affect the redox balance of the body, decreasing oxidative damage to lipids, and improving antioxidant status in humans.

### 3.5. Pearson’s Correlation

Pearson’s correlation indicates the degree of association between two variables that is, the change in magnitude in one variable is associated with the change in magnitude of another variable. There are positive and negative Pearson correlation coefficients that indicate an increase or decrease in another variable. The values of the coefficients can take values from −1 to 1, where zero indicates that there is no linear association between the tested variables, while 1 or −1 indicates a high association [57]. Table 4 shows the Pearson’s correlation coefficients for the evaluated responses; coefficients in bold indicate a statistically significant correlation (*p* ≤ 0.05). *Portulaca oleracea* presented a significant positive correlation with the content of total phenols, while *Chenopodium album* presented a significant negative correlation with DPPH (*p* ≤ 0.05); on the other hand, *Opuntia oligacantha* Förster presented a significant negative correlation with the content of total phenols and ABTS and a positive correlation with DPPH. The content of total phenols presented a positive correlation with ABTS, while the content of total flavonoids presented a negative correlation with DPPH and a positive correlation with ABTS.

## 4. Conclusions

According to the values obtained, the highest contents of total phenols, flavonoids, and antioxidant activity were observed in the mixtures where *Portulaca oleracea* and *Chenopodium album* were included. In the ternary mixtures where *Amaranthus tricolor* was present and *Portulaca oleracea* was not found, the lowest values for the evaluated responses were observed. The quaternary mixture did not present a synergistic effect on the evaluated responses. Pearson’s correlation supported that ABTS reacted both with total phenols and flavonoids. However, the presence of xoconostle within the mixtures can enrich the content of phenolic-type compounds, such as chlorogenic acid, phloridzin, and naringenin. Plant-based foods and their proposals in the form of mixtures are presented as viable alternatives for the generation of vegetable mixtures rich in phenolic compounds that can be used to fortify foods or even as food supplements. In this study, a mixture of *Portulaca oleracea* and *Chenopodium album* is recommended in a 1:1 ratio.

## Figures and Tables

**Table 2:** Estimated regression coefficients of the squared model adjusted for the responses evaluated.

Response	Linear				Interactions						R^2^
	*β*_1_ *X*_1_	*β*_2_ *X*_2_	*β*_3_ *X*_3_	*β*_4_ *X*_4_	*β*_1_ *β*_2_ *X*_1_ *X*_2_	*β*_1_ *β*_3_ *X*_1_ *X*_3_	*β*_1_ *β*_4_ *X*_1_ *X*_4_	*β*_2_ *β*_3_ *X*_2_ *X*_3_	*β*_2_ *β*_4_ *X*_2_ *X*_4_	*β*_3_ *β*_4_ *X*_3_ *X*_4_	
Total phenols	**9.96**	**10.15**	**7.16**	**7.24**	4.40	**9.40**	**11.62**	**−5.16**	**−7.55**	−0.02	0.93
Flavonoids	**8.91**	**17.54**	**2.53**	**15.22**	1.27	**13.27**	**5.40**	**−8.38**	**−13.80**	**−3.63**	0.99
DPPH	**24.90**	**21.49**	**27.71**	**22.33**	**8.51**	2.20	**6.51**	0.99	**8.66**	3.43	0.95
ABTS	**74.14**	**73.80**	**43.21**	**61.11**	**−26.91**	**42.33**	−15.65	**−78.00**	**−97.74**	**−47.45**	0.98

*X*_1_ = *Portulaca oleracea*; *X*_2_ = *Chenopodium album*; *X*_3_ = *Opuntia oligacantha* Förster var. Ulapa; *X*_4_ = *Amaranthus tricolor.* Numbers in bold indicate significant statistical effects on the evaluated response.

**Table 3:** Total phenols, total flavonoids, and antioxidant activity in experimental designs *X*_1_ (*Portulaca Oleracea*), *X*_2_ (*Chenopodium album*), *X*_3_ (*Opuntia oligacantha* Förster var. Ulapa), and *X*_4_ (*Amaranthus tricolor)*.

	Mixture	*X*_1_	*X*_2_	*X*_3_	*X*_4_	TPC (mg GAE g^−1^ DW)	TF (mg QE g^−1^ DW)	DPPH (µmol TE g^−1^ DW)	ABTS (µmol TE g^−1^ DW)
Individual components	1	1.0	0.0	0.0	0.0	10.1 ± 0.26 **^d^**	9.1 ± 0.18 **^c^**	24.9 ± 0.09 **^d^**	74.6 ± 0.25 **^l^**
	2	0.0	1.0	0.0	0.0	10.2 ± 0.31 **^d^**	17.6 ± 0.14 **^j^**	21.4 ± 0.09 **^a^**	74.2 ± 0.12 **^l^**
	3	0.0	0.0	1.0	0.0	7.3 ± 0.01 **^c^**	2.6 ± 0.03 **^a^**	27.7 ± 0.09 **^j^**	43.4 ± 0.39 **^e^**
	4	0.0	0.0	0.0	1.0	7.4 ± 0.28 **^c^**	15.4 ± 0.1 **^i^**	22.2 ± 0.04 **^b^**	61.6 ± 0.12 **^i^**
Binary mixture	5	0.5	0.5	0.0	0.0	11.1 ± 0.03 **^f^**	13.5 ± 0.03 **^h^**	25.5 ± 0.07 **^e^**	66.0 ± 0.07 **^j^**
	6	0.5	0.0	0.5	0.0	10.6 ± 0.06 **^e^**	8.8 ± 0.04 **^c^**	26.8 ± 0.07 **^i^**	68.9 ± 0.07 **^k^**
	7	0.5	0.0	0.0	0.5	11.3 ± 0.02 **^f^**	13.2 ± 0.02 **^g^**	25.5 ± 0.07 **^e^**	62.2 ± 0.43 **^i^**
	8	0.0	0.5	0.5	0.0	7.1 ± 0.05 **^b^**^,**c**^	7.9 ± 0.04 **^b^**	24.9 ± 0.09 **^d^**	38.7 ± 0.28 **^b^**
	9	0.0	0.5	0.0	0.5	6.6 ± 0.06 **^a^**	12.9 ± 0.05 **^f^**	24.4 ± 0.11 **^c^**	41.6 ± 0.19 **^d^**
	10	0.0	0.0	0.5	0.5	6.9 ± 0.01 **^a^**^,**b**^	7.8 ± 0.01 **^b^**	26.0 ± 0.11 **^f^**^,**g**^	39.8 ± 0.19 **^c^**
Ternary mixture	11	0.33	0.33	0.33	0.0	10.0 ± 0.04 **^d^**	10.4 ± 0.02 **^d^**	26.2 ± 0.11 **^g^**^,**h**^	56.3 ± 0.14 **^g^**
	12	0.33	0.33	0.0	0.33	9.9 ± 0.04 **^d^**	13.1 ± 0.03 **^g^**	25.1 ± 0.04 **^d^**	56.2 ± 0.19 **^g^**
	13	0.33	0.0	0.33	0.33	10.7 ± 0.04 **^e^**	10.8 ± 0.03 **^e^**	26.3 ± 0.07 **^h^**	57.3 ± 0.14 **^h^**
	14	0.0	0.33	0.33	0.33	6.9 ± 0.03 **^a^**^,**b**^	8.9 ± 0.03 **^c^**	25.1 ± 0.07 **^d^**	34.4 ± 0.26 **^a^**
Quaternary mixture	15	0.25	0.25	0.25	0.25	10.2 ± 0.05 **^d^**	10.9 ± 0.04 **^e^**	25.9 ± 0.04 **^f^**	51.3 ± 0.12 **^f^**

Different letters in each column represent a statistically significant difference according to the Tukey test (*p* ≤ 0.05); the lowest letter represents the highest value. TPC: total phenolic content; TF: total flavonoid content; GAE: gallic acid equivalents; QE: quercetin equivalents; TE: Trolox equivalents; DW: dry weight of raw material.

**Table 4:** Pearson’s correlation coefficients for the responses evaluated.

	Total Phenol Content	Total Flavonoid Content	DPPH	ABTS
**Total Phenol Content**	**1**	0.37	0.10	**0.78**
**Total Flavonoid Content**		**1**	**−0.79**	**0.52**
**DPPH**			**1**	−0.31
**ABTS**				**1**

## Data Availability

All data presented in this study are available and are included in this published article.

## Supplementary Materials

The following supporting information can be downloaded at https://www.mdpi.com/article/10.3390/foods12183479/s1, Table S1: Phenolic acids and flavonoids identified by HPLC in *O. oligacantha* (Förster) var. Ulapa. References [58,59,60,61] cited.
