# Supplementary material for: Application of a Simplex–Centroid Mixture Design to Evaluate the Phenolic Compound Content and Antioxidant Potential of Plants Grown in Mexico
Source: Foods. 2023 Sep 19;12(18):3479. doi: 10.3390/foods12183479 (PMC10529064; doi:10.3390/foods12183479)
Supplement: Supplementary file 1 [file foods-12-03479-s001.zip › foods-2585275-supplementary.pdf]

SUPPLEMENTARY MATERIAL

Table S1. Phenolic acids and flavonoids identified by HPLC in *O. oligacantha* (Förster) var. Ulapa.

**Phenolic acids** (μg/g DW)		*Opuntia joconostle* (Pericarp and meso-carp)[58]	*Opuntia joconostle* F.A.C. Weber ex Diguet var. Cuaresmeño (Epicarp)[59]	*Opuntia matudae* var. Cuaresmeño (Pericarp)[60]	*Opuntia matudae* Scheinvar var. Rosa (Epicarp)[61]
**Gallic**	534.11 ± 0.05			*	
**Chlorogenic**	2305.66 ± 0.07				
**Syringic**	265.53 ± 0.10	*			
**Vanillic**	ND	*		*	
***p*-hydroxybenzoic**	ND				
**Caffeic**	ND	*			*
**Ferulic**	207.33 ± 0.02		*		*
***p*-coumaric**	ND				
Total	3312.63				

(Cont.)

**Flavonoids** (µg/g DW)		*Opuntia joconostle* (Pericarp and mesocarp)[58]	*Opuntia joconostle* F.A.C. Weber ex Diguet var. Cuaresmeño (Epicarp)[59]	*Opuntia stricta* Haw; *undulata* Griff; *ficus indica* L. (All parts of the fruit)[50]	*Opuntia matudae* Scheinvar var. Rosa (Epicarp)[61]
**Apigenin**	ND				
**Rutin**	437.68 ± 0.01				
**Phloridzin**	2674.36 ± 0.12				
**Myricetin**	ND				
**Quercetin**	ND	*	*	*	*
**Naringenin**	1143.40 ± 0.06				
**Phloretin**	ND				
**Galangin**	88.43 ± 0.17				
Total	4343.87				

The table presents data of the mean + SD (n=3) for the results found in this research in the samples of *Opuntia oligacantha* (Förster) var. Ulapa; * Presence of phenolic acids and flavonoids identified in previous research for genera *Opuntia* spp; **ND**: not detected; **DW**: dry weight of raw material.
